# Machine learning approaches for tuberculosis prevalence and risk factor association among high-risk groups in Kigali health facilities

**DOI:** 10.21203/rs.3.rs-10453356/v1

**Published:** 2026-07-27

**Authors:** Theophilla Igihozo, Fiacre Rugamba Rugero, Emma Marie Umutoniwase, Emelie Ndoli, Emmanuel Niyonshuti, Rwubaka Eugene, Emmanuel Sibomana, Melissa Uwase, Dieudonne Kayiranga, Michael Mugisha, Celestin Twizere, Isambi Sailon Mbalawata, Davila-Roman Victor, Adams Wilcox, Randi Foraker, Isaac Gomina, Michelle Schneider, David Tumusiime, Emmy Mugisha

**Affiliations:** College of Medicine and Health Sciences, University of Rwanda; College of Medicine and Health Sciences, University of Rwanda; College of Medicine and Health Sciences, University of Rwanda; College of Medicine and Health Sciences, University of Rwanda; College of Medicine and Health Sciences, University of Rwanda; College of Medicine and Health Sciences, University of Rwanda; College of Business and Economics, University of Rwanda; College of Medicine and Health Sciences, University of Rwanda; College of Medicine and Health Sciences, University of Rwanda; College of Medicine and Health Sciences, University of Rwanda; Health Informatics, Regional Center of Excellence in Biomedical Engineering & E-Health, University of Rwanda, Kigali Innovation City; African Institute for Mathematical Sciences Research and Innovation Centre; School of Medicine, Washington University in St. Louis, St. Louis; School of Medicine, Washington University in St. Louis, St. Louis; School of Medicine, University of Missouri, Columbia; School of Medicine, University of Missouri, Columbia; Institute for Informatics, Data Science and Biostatistics, Washington University in St. Louis, St. Louis; College of Medicine and Health Sciences, University of Rwanda; Health Informatics, Regional Center of Excellence in Biomedical Engineering & E-Health, University of Rwanda, Kigali Innovation City

**Keywords:** Tuberculosis, Machine Learning, Electronic Medical Records, Risk Factors

## Abstract

**Background:**

Tuberculosis (TB) remains a major global health challenge, disproportionately affecting vulnerable populations in resource-limited settings. In Rwanda, the burden is high among high-risk groups including prisoners, mining workers, people living with HIV, and healthcare workers. Despite well-established national surveillance infrastructure, published evidence on applying machine learning (ML) to routinely collected electronic medical records (EMR) for identifying factors associated with TB in these populations remains limited. This study develops and compares multiple ML models to identify factors associated with TB among high-risk groups using national surveillance data from Kigali health facilities, while emphasizing careful feature engineering to avoid information leakage.

**Methods:**

We conducted a retrospective, cross-sectional analysis of 2,254 high-risk patient EMR from Rwanda Biomedical Center (RBC) surveillance systems, with TB status defined by GeneXpert results. Rigorous data cleaning and preprocessing were performed, including exclusion of records with indeterminate outcomes, removal of diagnostic and operational variables that could introduce information leakage, imputation of missing values, normalization of numerical features, and one-hot encoding of categorical variables. Multiple supervised ML models were developed and evaluated using a stratified train–test split, including logistic regression (LR), balanced random forest (BRF), gradient boosting methods (XGBoost, LightGBM, and GBM), support vector machines (SVM) with a radial basis function kernel, and an EasyEnsemble classifier. Model performance was assessed using accuracy, precision, recall, F1-score, and area under the receiver operating characteristic (ROC) curve (AUC).

**Results:**

After excluding indeterminate GeneXpert outcomes, 358 cases were retained for evaluation. Ensemble-based models showed consistently strong and stable performance, with BRF achieving the highest discrimination (AUC = 0.86), while GBM, SVM, and EasyEnsemble classifiers showed comparable performance across accuracy, precision, recall, and F1-score. Feature-importance analysis identified clinically meaningful factors, associated with TB, including site of disease, age, TB-related body mass index, HIV status, and diabetes status, indicating that the models relied on plausible biological and demographic risk factors rather than diagnostic artifacts.

**Conclusions:**

This first application of EMR-driven ML for identifying TB risk factor associations in Rwanda shows that ensemble models, combined with rigorous leakage control, achieve promising performance among high-risk populations. These tools could support national TB surveillance and screening to enhance early detection and resource allocation.

## Background

Tuberculosis (TB) remains one of the leading causes of death from infectious diseases worldwide, with the greatest burden concentrated in low- and middle-income countries [1]. In 2024, an estimated 10.7 million people fell ill with TB and 1.23 million died from the disease [2]. Rwanda has made notable progress in TB control but continues to face persistent challenges, particularly among populations classified as high-risk, including prisoners, mining workers, refugees, health facility staff, and individuals living with human immunodeficiency virus (HIV) infection [3, 4]. Comorbidities such as diabetes mellitus and HIV infection further exacerbate susceptibility by accelerating progression from latent to active TB disease [5].

The expansion of digital health systems across sub-Saharan Africa presents new opportunities to strengthen TB detection and surveillance. Rwanda, in particular, has invested heavily in electronic medical records (EMR) and nationwide diagnostic reporting infrastructure. These systems contain rich clinical, demographic, and programmatic data that, if systematically analyzed, could provide actionable insights for TB prevention and control. At the same time, machine learning (ML) has rapidly emerged as a powerful tool capable of identifying complex patterns in health data, improving disease prediction, and supporting clinical and public-health decision-making [6].

While several ML applications have been developed for TB detection, most existing studies have focused on radiological images, sputum data, or genomic sequencing [7], with comparatively limited use of routine EMR data in the African context. Importantly, published evidence on the application of ML approaches for identifying factors associated with a TB diagnosis using routine EMR data from national surveillance systems remains limited in Rwanda, despite the availability of comprehensive national data streams.

National TB surveillance data from RBC annual reports (2014–2015 to 2023–2024) [8–16] reveal a persistent TB/HIV burden and a marked increase in TB notifications among high-risk groups in recent years, underscoring the need for a more targeted, data-driven approach to identifying potential co-morbid conditions.

While neighboring East African countries such as Kenya, Uganda, and Tanzania have begun incorporating ML techniques into various aspects of TB research, including diagnostic prediction, treatment adherence, and antimicrobial resistance modeling [17]-[22], no published ML-based TB studies using routine EMR data have been identified in Rwanda.

This study therefore develops and compares multiple ML models to evaluate factors associated with TB among high-risk groups, and to assess model performance under real-world screening conditions using national surveillance data from Kigali health facilities.

## Methods

### Data source and study design

The data used in this study were provided by RBC, the national authority responsible for implementing health research and disease surveillance. The dataset comprised records from various health facilities in Kigali, Rwanda’s capital, collected between January 2024 and March 2025. The RBC electronic system is the primary platform used to manage TB patient data across the country, and it includes information on diagnostics, patient demographics, comorbidities, and risk exposures.

#### Study population and variables

The study included 2,254 patients who underwent TB screening and were categorized as belonging to high-risk groups. High-risk classification was based on factors such as HIV infection, history of exposure to TB, being a refugee, prisoner, mining worker, or health facility staff. The outcome variable was GeneXpert-confirmed TB status (Detected vs. Not Detected).

Independent variables included age, sex, HIV status, diabetic status, refugee status, contact with known TB-positive individuals, facility of diagnosis, and year of visit. Additional EMR features reflecting clinical status, history of TB, and other comorbidities were included where data availability permitted.

#### Data cleaning and preprocessing

Data preprocessing steps were conducted in Python (version 3.10.16) and are described below, with particular attention to preventing information leakage and improving model generalizability.

Duplicate records were removed, and categorical variables were standardized to correct inconsistencies in spacing and letter case. A systematic assessment of missingness was performed, and variables with more than 30% missing values primarily laboratory resistance tests, culture results, and administrative dates were excluded due to limited availability and potential bias.

Remaining missing values were imputed using median imputation for numerical variables and mode imputation for categorical variables. To prevent data leakage and artificial inflation of model performance, all variables that directly reflected TB diagnostic outcomes, laboratory confirmation dates, or post-diagnosis administrative information (e.g., GeneXpert results, smear and culture results, diagnostic dates, and confirmation methods) were explicitly removed prior to model development.

Additional non-informative identifiers and redundant variables (e.g., patient IDs, duplicate age fields, and components already used to compute body mass index (BMI)) were also excluded.

Categorical predictors were transformed using one-hot encoding with reference category dropping to avoid multicollinearity, while key continuous variables (current age and TB-related BMI) were normalized using standard scaling. The final feature matrix consisted exclusively of pre-diagnostic demographic, clinical, and risk-factor variables suitable for analysis. Stratified train–test splitting was applied to preserve the original class distribution and mitigate learning bias during model training and evaluation.

#### Risk group analysis

Statistical analyses were performed to determine the relationship between high-risk variables and TB status. Chi-square tests were used to evaluate associations between categorical variables and TB status, and two sample t-tests assessed mean differences in continuous variables such as age.

TB prevalence was analyzed within each risk group using Table 1. These analyses informed the feature engineering process by highlighting potentially relevant variables.

#### Model development and evaluation

A total of seven supervised ML classifiers were developed and evaluated: LR, BRF, XGBoost, support vector machine with a radial basis function kernel (SVM-RBF), LightGBM, gradient boosting machine (GBM), and EasyEnsemble. Models were trained on approximately 85% of the cleaned dataset and evaluated on a held-out stratified test subset (15%, *n* = 358) to preserve the original distribution of TB outcomes. To address class imbalance in the training data, Synthetic Minority Over-sampling Technique (SMOTE) resampling was applied prior to model fitting. Hyperparameters for each model were optimized using grid search with cross-validation on the training set. Model performance was assessed using accuracy, precision, recall, F1-score, and the area under the receiver operating characteristic (ROC) curve (AUC). Confusion matrices and ROC curves were generated to evaluate classification behavior and trade-offs between sensitivity and specificity.

## Results

### Study population characteristics

The distribution of patients by year of diagnosis shows a sharp increase in 2024, with 1,598 cases, accounting for much of the dataset. This is followed by 656 cases in 2025, indicating a decline compared to the previous year. This highlights a concentrated rise in diagnoses beginning in 2024, suggesting a possible increase in screening activities, improved data reporting, or a true rise in disease prevalence during that period.

The gender distribution indicated that males accounted for approximately 70% of cases. This male predominance is consistent with tuberculosis notification patterns reported in Rwanda, where higher case detection among men has been linked to a combination of behavioral, occupational, and health-seeking factors. In the present dataset, which was collected through RBC surveillance systems with a focus on high-risk groups (HRGs), men may be disproportionately represented due to greater involvement in occupations and social settings associated with increased TB exposure, including work in mining, construction, and other high-density environments. In addition, delayed healthcare-seeking behavior among men may lead to more advanced disease at the time of presentation, increasing the likelihood of bacteriological confirmation. Similar male-to-female ratios have been documented in national TB surveillance reports, supporting the plausibility of the observed distribution in this dataset.

**Figure 1:**
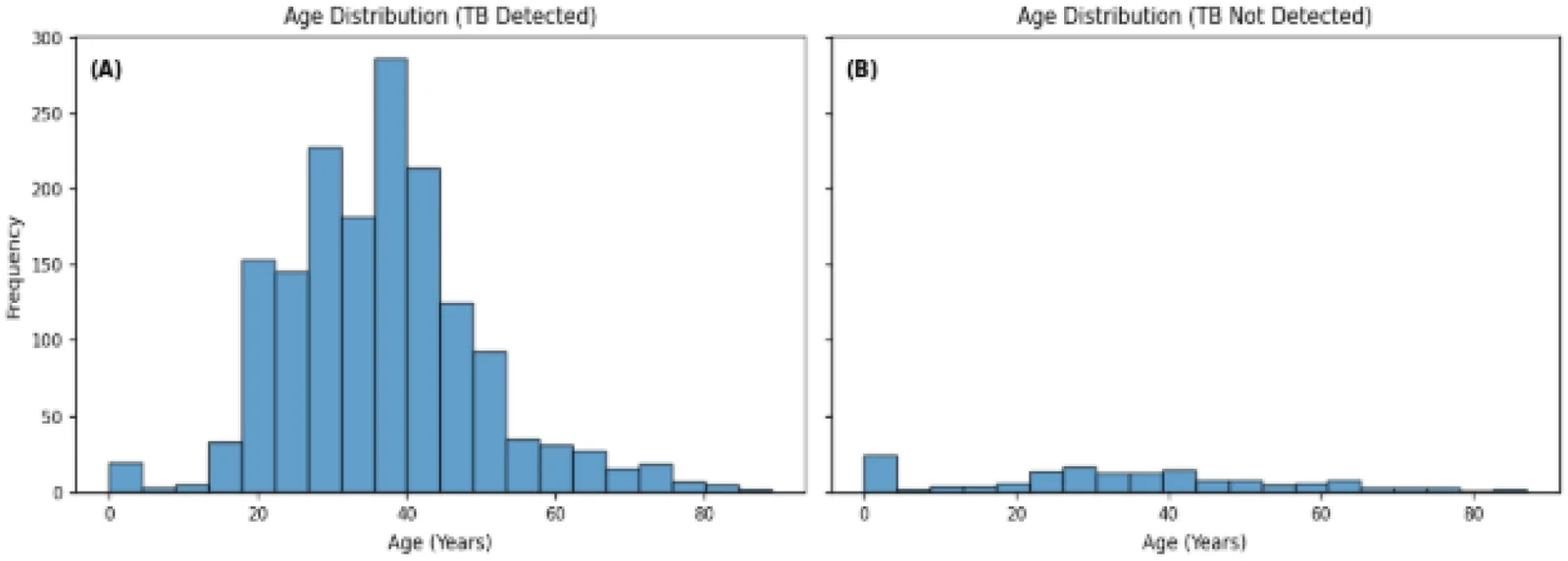
Age distribution of individuals with GeneXpert-confirmed tuberculosis (A) and GeneXpert-negative results (B) in the study population

Figure 1 presents the age distribution of individuals with GeneXpert-confirmed tuberculosis (A) and those with GeneXpert-negative results (B). Among TB-positive individuals, cases were most common among adults aged 20–45 years, with a pronounced peak around the 30–40 age group. A smaller number of cases were observed among young children, followed by a gradual decline in frequency with increasing age. In contrast, the non-TB group showed a smaller and more dispersed age distribution, reflecting the lower number of individuals with GeneXpert-negative results in this surveillance dataset. Overall, these findings indicate that TB affects a broad age range but is particularly concentrated among economically active adults. In the full study population (N = 2,254), 91.4% of individuals had TB recorded as detected by GeneXpert, consistent with the class distribution summarized in Table 1.

#### Risk factor associations

**Figure 2:**
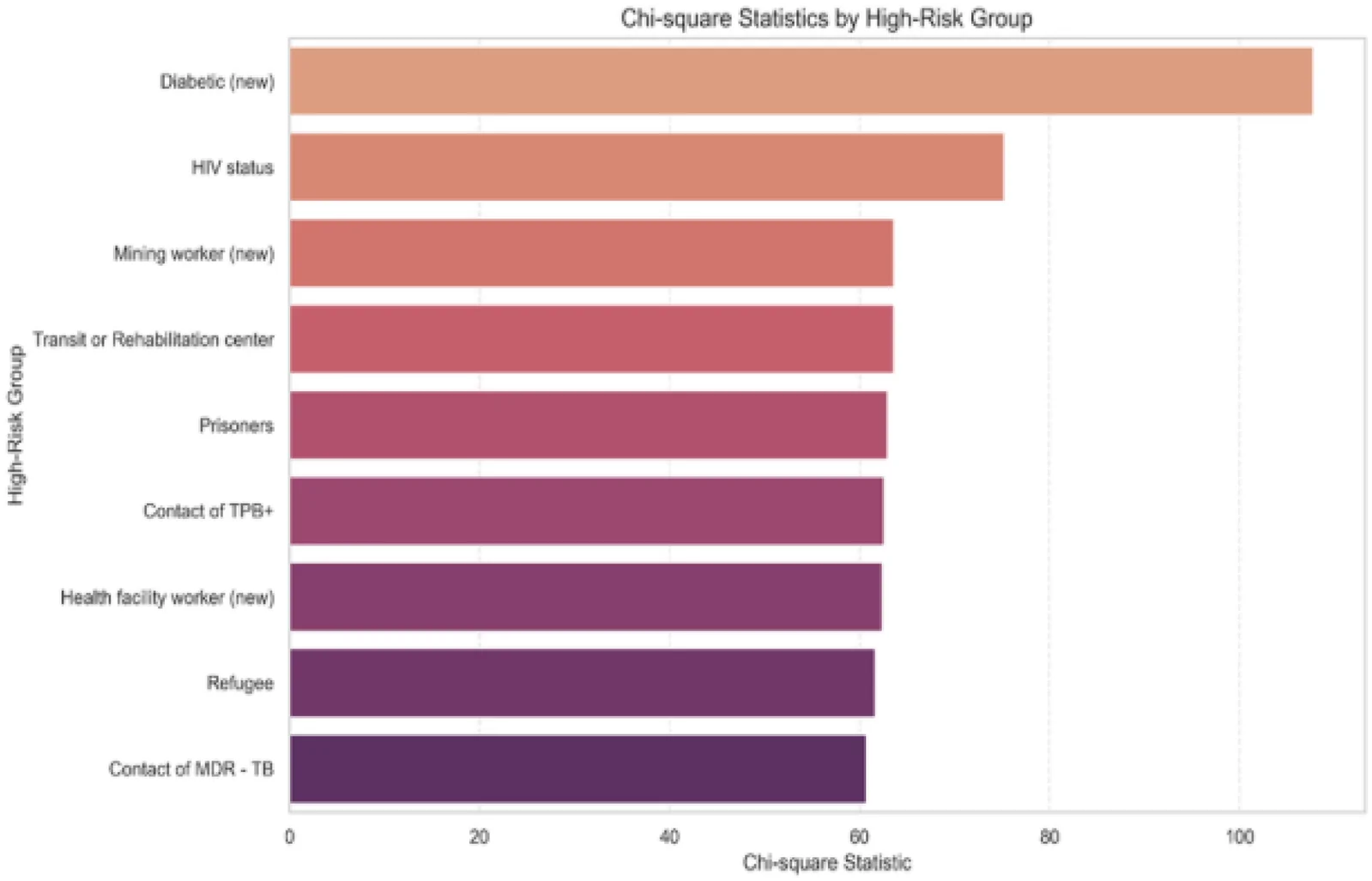
Chi-square statistics by high-risk group

Statistically significant associations were observed between TB status and multiple risk indicators (p < 0.05). Mining workers, prisoners, and refugees exhibited the highest TB prevalence, followed by individuals living with HIV and health facility workers. Figure 2 summarizes the Chi-square test results, illustrating the strength of association between TB detection and various high-risk categories. The highest Chi-square statistics were observed among diabetic individuals, indicating a strong association with TB. HIV-positive individuals, mining workers, and those in rehabilitation centers also demonstrated significantly elevated TB rates. Other high-risk groups, such as prisoners, health facility workers, and refugees, showed moderate but still statistically significant associations. These findings highlight the need for targeted interventions among these populations to reduce TB transmission and improve case detection.

Table 1 summarizes the distribution of GeneXpert MTB results across all 2,254 individuals categorized into high-risk groups. Across nearly all categories, the proportion of TB-positive (Detected) results exceeded 90%, reflecting the very high pre-test probability in this high-risk cohort. Prisoners with reported incarceration (“Yes”), refugees (“Yes”), and diabetic patients with unknown diabetic status showed some of the highest detection proportions, with values of 95%, 100%, and 99%, respectively. In contrast, diabetic patients with confirmed diabetes (“Yes”) had a lower detection proportion of 85%. Mining workers demonstrated the greatest variability, with those categorized as exposed (“Yes”) exhibiting the lowest detection rate (78%) and the highest proportion of non-detected results (22%). Contacts of MDR-TB also showed modest variability (88% detected; 12% not detected), whereas health facility workers and contacts of TPB+ (TB-positive person) maintained consistently high detection proportions.

Importantly, individuals whose GeneXpert outcome was recorded as “Not done” or “No result” were excluded from all ML analyses, as these incomplete diagnostic outcomes do not provide a definitive TB status. Including such records would introduce misclassification bias and reduce the validity of supervised learning approaches that require a clearly defined outcome label. These exclusions ensured that the models were trained and evaluated only in cases with confirmed TB status, strengthening the reliability of the association findings.

### ML model performance

Table 2 summarizes the performance of all evaluated models on the stratified test subset (n = 358), which preserved the original class imbalance of the cohort. Overall, all models demonstrated strong performance, with consistently high precision and recall for tuberculosis detection, indicating reliable identification of TB-positive cases across different algorithmic approaches.

LR served as a baseline model, achieving an accuracy of 0.82 and a recall of 0.85. Although its performance was acceptable, its lower AUC (0.77) suggests limited discriminative capacity compared with more advanced methods. In contrast, all ensemble-based models showed clear and consistent improvements across most evaluation metrics.

F1-score (0.93), indicating the most favorable balance between sensitivity and specificity among the evaluated models. SVM with an RBF kernel also demonstrated strong performance, achieving an AUC of 0.84 and an F1-score of 0.92, reflecting its ability to capture non-linear relationships in the data.

Gradient-boosting–based methods, including XGBoost, LightGBM, and GBM, showed highly comparable and stable performance, with accuracies ranging from 0.85 to 0.88 and F1-scores consistently above 0.91. EasyEnsemble likewise performed competitively, particularly in terms of precision (0.96) and recall (0.86), further confirming the robustness of ensemble learning approaches for this task.

**Figure 3:**
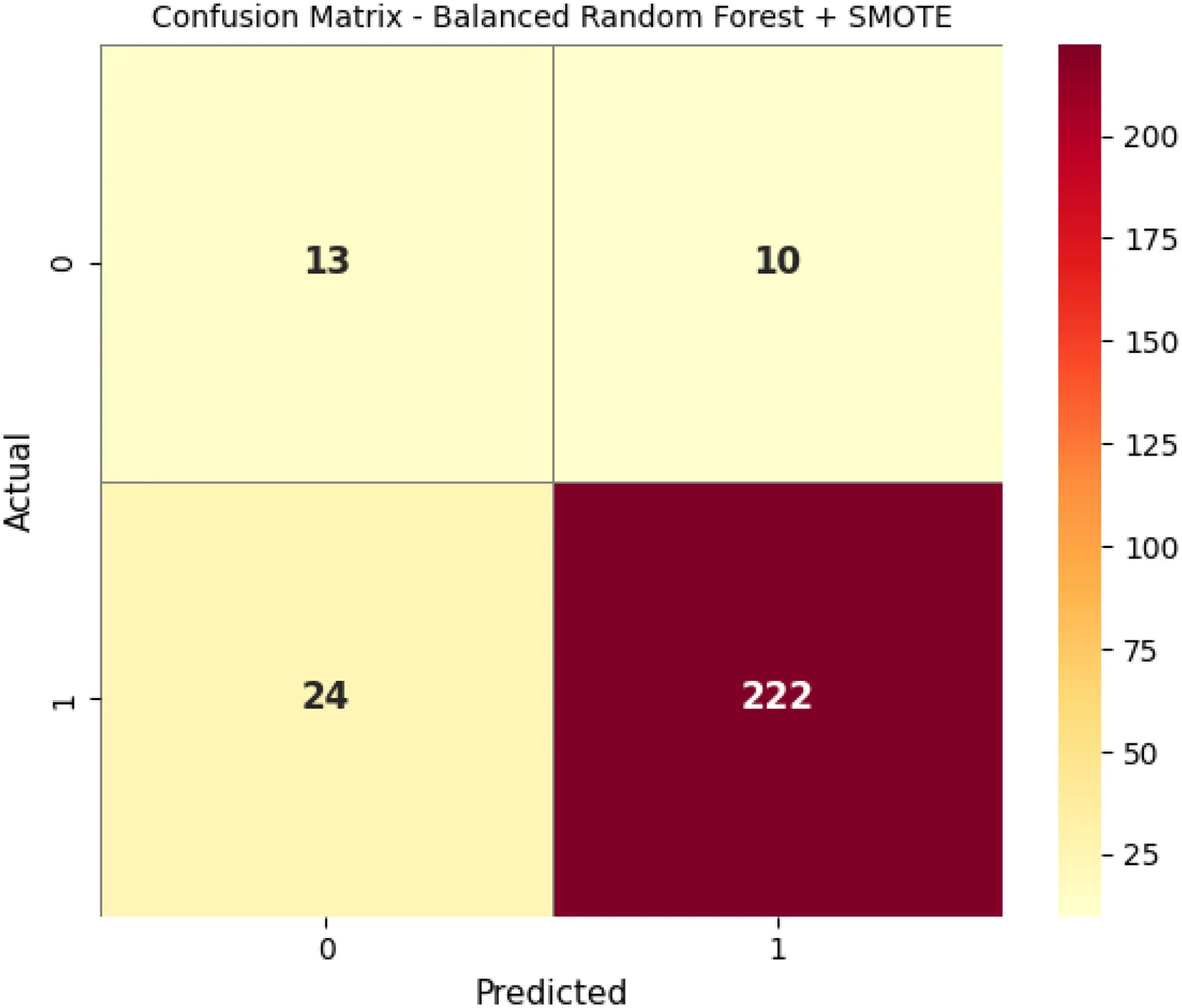
Confusion matrix of BRF model

**Figure 4:**
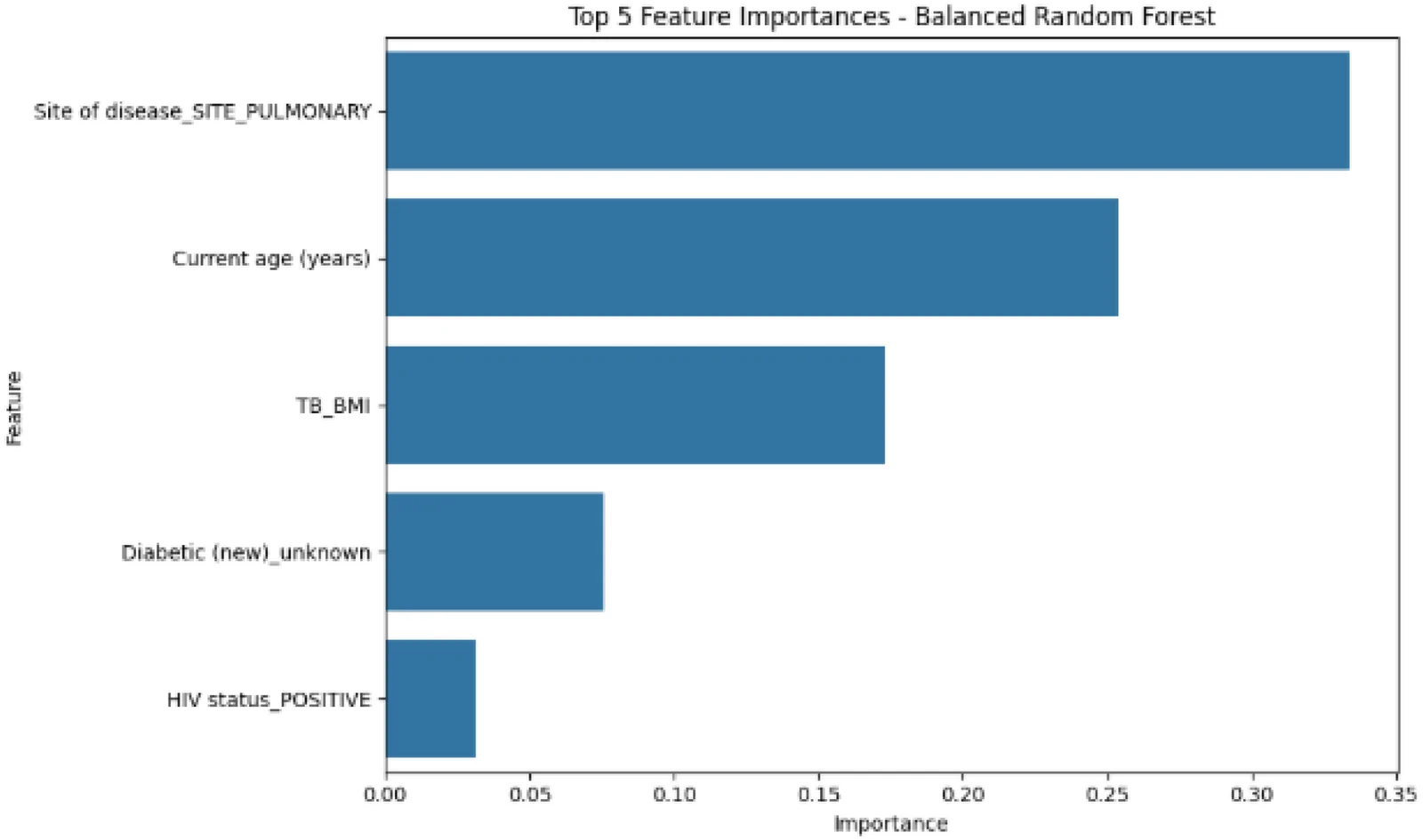
Top five feature importances derived from the BRF model after feature-selection and leakage control

Figure 3 presents the confusion matrix of the BRF model, where class 1 corresponds to *GeneXpert MTB Detected* cases and class 0 corresponds to *GeneXpert MTB Not Detected* cases. Figure 4 compares the receiver operating characteristic (ROC) curves of all evaluated models, which show consistently strong discrimination performance, with AUC values ranging from 0.77 to 0.86.

The task in this study focuses on classifying bacteriological TB detection status (Detected vs. Not Detected) within a clinically enriched cohort derived from RBC surveillance data, rather than population-level TB diagnosis. The stratified test subset preserved the original class distribution, in which TB-detected cases (class 1) constituted approximately 91% of records, contributing to high overall accuracy across models.

### Feature importance analysis

**Figure 5:**
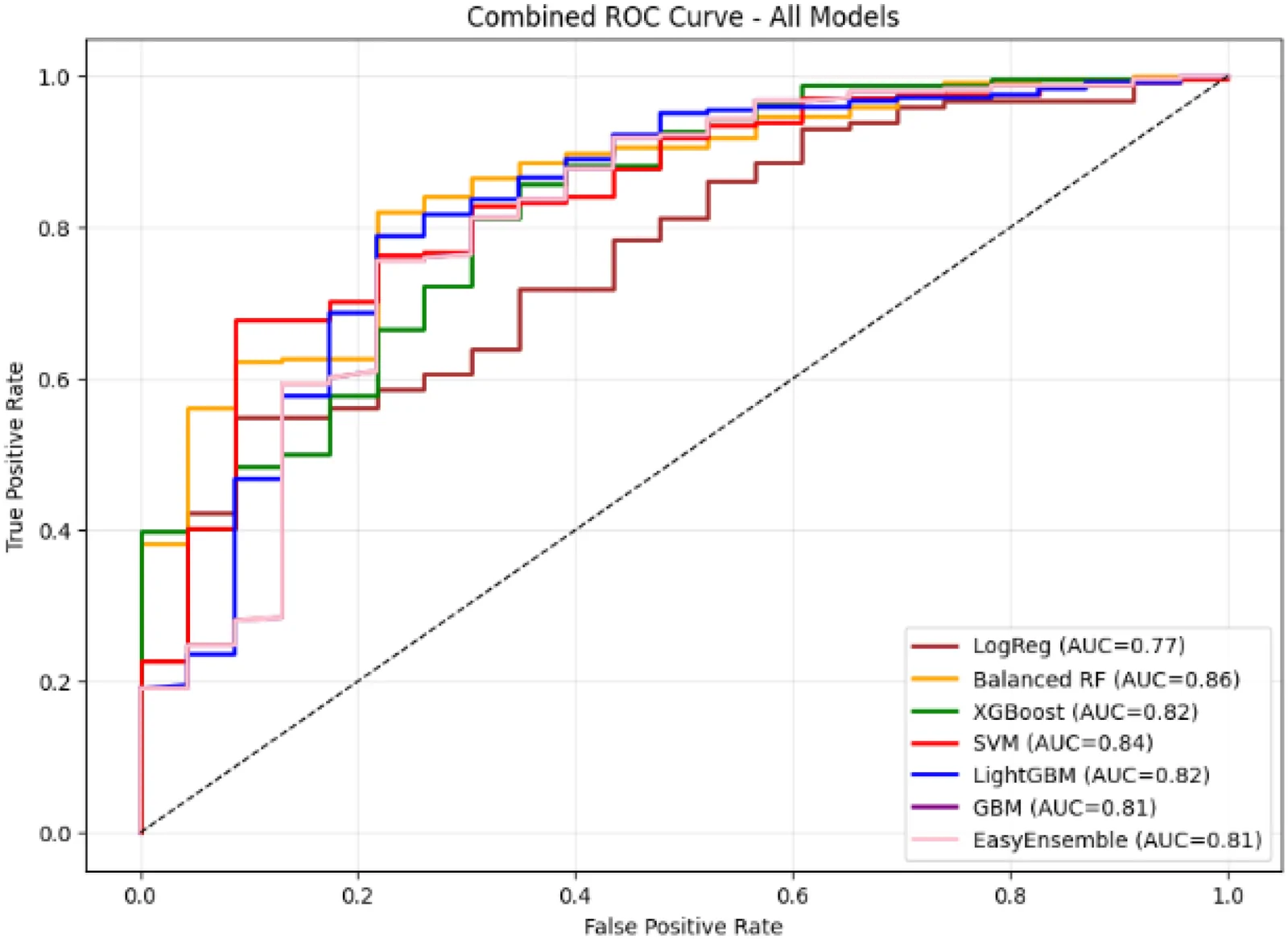
Receiver operating characteristic (ROC) curves comparing the performance of all machine learning models for classifying GeneXpert MTB detection status

Feature-importance analysis using the BRF model (Fig. 5) highlighted several clinically meaningful factors associated with TB classification after removal of diagnostic and operational variables. The most influential feature was pulmonary disease site, reflecting the higher likelihood of bacteriological confirmation among pulmonary TB cases. Current age and TB-related body mass index were also among the strongest contributors, consistent with established associations between age, nutritional status, and TB. Additional contributing factors included diabetes status and HIV-positive status, both of which are known to increase susceptibility to TB and influence disease presentation. Overall, the feature-importance profile suggests that the model relied on biologically and clinically interpretable characteristics rather than artifacts of the diagnostic process.

## Discussion

### Principal findings

This study demonstrates the feasibility and effectiveness of applying multiple ML models to detect associations of risk factors with TB status among high-risk groups using routinely collected EMR data in Rwanda. After careful feature engineering and mitigation of information leakage, ensemble tree-based models consistently outperformed linear approaches, with BRF and GBM based models showing the strongest and most stable performance across evaluation metrics. Feature-importance analysis revealed that clinically meaningful variables, including site of disease, current age, TB-related BMI, HIV status, and diabetes status, were key contributors to model outputs, aligning well with established TB epidemiology.

To our knowledge, published evidence on the application of ML approaches for evaluating risk factor associations with TB using routine EMR data from national surveillance systems remains limited in Rwanda, and this study represents a significant contribution toward addressing this underexplored area. These findings demonstrate that carefully curated clinical data streams, when coupled with appropriate feature engineering and model selection, can support risk stratification and targeted screening among high-risk populations, thereby complementing existing TB control strategies.

### Interpretation and comparisons

The limited published ML-based TB research in Rwanda underscores the significance of these findings, particularly as neighboring countries have begun incorporating similar approaches into TB research. Our findings align with recent global studies showing that individuals with HIV and diabetes are associated with a higher prevalence of TB. Diabetes increases the risk of active TB by approximately two-to-three times compared to people without diabetes [23]. Similarly, TB is the leading cause of death among people living with HIV, who may be up to 18 times more likely to develop TB compared to HIV-uninfected individuals [24]. The high TB burden observed in incarcerated and other crowded environments further supports longstanding evidence that overcrowded and poorly ventilated settings drive TB transmission [25]. Unlike previous machine learning applications that primarily utilize radiological data, our study demonstrates the feasibility and effectiveness of using structured EMR for identifying TB risk factor associations. This approach is particularly valuable in resource-limited settings such as Rwanda and sub-Saharan Africa, where access to radiological tools may be limited.

### Implications for policy and practice

The integration of ML models into Rwanda’s TB screening infrastructure holds significant potential to enhance clinical decision-making and inform public health strategies. These models can assist in prioritizing high-risk individuals for diagnostic testing, particularly in resource-limited settings where efficient allocation of services is critical. Furthermore, incorporating ML tools into existing health information systems—such as the Health Management Information System (HMIS) and Treatment and Research Acquired Immunodeficiency Syndrome (AIDS) Center network (TRACnet) is operationally feasible, provided that appropriate investments in capacity building, training, and technical infrastructure are made.

### Strengths and limitations

This study has several notable strengths. First, it leverages a large, national-level EMR dataset collected through RBC surveillance systems, providing real-world clinical data from high-risk populations rather than curated research cohorts. To our knowledge, this is the first study in Rwanda to systematically apply and compare multiple ML models using routine national EMR data for TB assessment. Second, the study conducts a comprehensive comparison of multiple ML approaches using a stratified test subset, enabling a robust assessment of classification performance across different algorithmic families.

Several limitations should also be acknowledged. The retrospective nature of EMR data introduces potential biases related to clinical documentation practices and missing or inconsistently recorded variables. Although we explicitly removed features that could directly or indirectly encode TB testing or diagnostic status, residual correlations inherent to routine care data cannot be completely excluded. In addition, because the study population was enriched for individuals at high risk of TB, with a high pre-test probability of a positive result, the exposures examined are not independent of the sampling process. Consequently, the models describe associations between characteristics and TB status within this selected high-risk cohort rather than predicting TB risk in a general population, and performance metrics should be interpreted in that context. The dataset also lacks radiographic information and key social determinants of health (e.g., income, housing conditions, or occupational exposure), which may further improve risk stratification if available. Finally, model performance was evaluated on an internal test subset using cross-sectional data; prospective validation and external evaluation in independent settings are needed before clinical deployment.

Overall, while these limitations warrant caution, the revised analyses demonstrate that ML models can identify associations when trained on carefully curated EMR data, supporting their potential role as decision-support tools for TB risk assessment among high-risk populations.

## Conclusions

This study demonstrates that ML algorithms can detect associations with TB using routine EMR collected by RBC among high-risk populations in Kigali. To our knowledge, this is the first application of EMR-driven ML methods for TB assessment in Rwanda, addressing an unmet need in the national TB research landscape.

Across the evaluated models, the BRF with SMOTE showed the strongest overall discrimination performance, while other ensemble approaches also achieved consistently strong results. These findings suggest that, after careful feature engineering to remove potential leakage variables and standardize features, EMR-based models can provide a promising tool for risk stratification and targeted screening support. With prospective validation and evaluation in routine implementation settings, such models could complement Rwanda’s TB surveillance and control strategies by helping prioritize high-risk individuals for timely testing and follow-up, particularly in resource-limited contexts.

## Figures and Tables

**Table 1:** Distribution of GeneXpert MTB results across high-risk groups in the study cohort

High-risk group	Category	n	Detected n (%)	Not detected n (%)
HIV status	Negative	1440	91	9
	Positive	347	92	8
Prisoner	No	1744	91	9
	Yes	44	95	5
Diabetic	No	1517	90	10
	Unknown	257	99	1
	Yes	13	85	15
Health facility worker	No	1773	91	9
	Yes	14	93	7
Refugee	No	1780	91	9
	Yes	7	100	0
Mining worker	No	1778	91	9
	Yes	9	78	22
Transit/rehabilitation center	No	1774	91	9
	Yes	13	100	0
Contact of MDR - TB	No	1779	91	9
	Yes	8	88	12
Contact of TPB+	No	1682	91	9
	Yes	105	93	7

**Table 2:** Model evaluation metrics computed on the stratified test subset (n = 358) after excluding records with indeterminate GeneXpert outcomes.

Model	Accuracy	Precision	Recall	F1-score	AUC
LR	0.82	0.95	0.85	0.9	0.77
BRF	0.87	0.96	0.9	0.93	0.86
XGBoost	0.87	0.95	0.91	0.93	0.82
SVM (RBF)	0.86	0.95	0.89	0.92	0.84
LightGBM	0.85	0.96	0.87	0.91	0.82
GBM	0.88	0.95	0.91	0.93	0.81
EasyEnsemble	0.84	0.96	0.86	0.91	0.81

## Data Availability

The datasets generated and/or analyzed during the current study are not publicly available due to legal and institutional restrictions concerning the confidentiality of health records in Rwanda. However, the data may be made available by the corresponding author (Theophilla Igihozo, igihozot2@gmail.com) upon reasonable request and with prior approval from the Rwanda National Ethics Committee.

## Abbreviations

AIDS: Acquired Immunodeficiency Syndrome
AUC: Area Under the Curve
BMI: Body Mass Index
BRF: Balanced Random Forest
EMR: Electronic Medical Record
GBM: Gradient Boosting Machine
HIV: Human Immunodeficiency Virus
HMIS: Health Management Information System
MDR: Multidrug-resistant
HRGs: High-Risk Groups
LR: Logistic Regression
ML: Machine Learning
MTB: Mycobacterium tuberculosis
RBC: Rwanda Biomedical Center
RBF: Radial Basis Function
ROC: Receiver Operating Characteristic
SMOTE: Synthetic Minority Over-sampling Technique
SVM: Support Vector Machine
TB: Tuberculosis
TRACnet: Treatment and Research AIDS Center network
